# Industrial-scale production and purification of a heterologous protein in *Lactococcus lactis *using the nisin-controlled gene expression system NICE: The case of lysostaphin

**DOI:** 10.1186/1475-2859-4-15

**Published:** 2005-05-27

**Authors:** Igor Mierau, Peter Leij, Iris van Swam, Barry Blommestein, Esther Floris, James Mond, Eddy J Smid

**Affiliations:** 1 NIZO food research, P.O. Box 20, 6710 BA Ede, The Netherlands; 2 Biosynexus, Inc., 9119 Gaither Road, Gaithersburg, MD 20877, USA

## Abstract

**Background:**

The NIsin-Controlled gene Expression system NICE of *Lactococcus lactis *is one of the most widespread used expression systems of Gram-positive bacteria. It is used in more than 100 laboratories for laboratory-scale gene expression experiments. However, *L. lactis *is also a micro-organism with a large biotechnological potential. Therefore, the aim of this study was to test whether protein production in *L. lactis *using the NICE system can also effectively be performed at the industrial-scale of fermentation.

**Results:**

Lysostaphin, an antibacterial protein (mainly against *Staphylococcus aureus*) from *S. simulans *biovar. Staphylolyticus, was used as a model system. Food-grade lysostaphin expression constructs in *L. lactis *were grown at 1L-, 300-L and 3000-L scale and induced with nisin for lysostaphin production. The induction process was equally effective at all scales and yields of about 100 mg/L were obtained. Up-scaling was easy and required no specific effort. Furthermore, we describe a simple and effective way of downstream processing to obtain a highly purified lysostaphin, which has been used for clinical phase I trials.

**Conclusion:**

This is the first example that shows that nisin-regulated gene expression in *L. lactis *can be used at industrial scale to produce large amounts of a target protein, such as lysostaphin. Downstream processing was simple and in a few steps produced a highly purified and active enzyme.

## Background

*Lactococcus lactis *is a Gram-positive bacterium that is widely used in food production such as cheese and butter manufacturing [1]. In the last two decades the physiology and genetics of this bacterium have been thoroughly characterized [2]. At present several genomes are either completely sequenced or close to completion [3,4]. Furthermore, *L. lactis *is easily genetically accessible and a wide variety of genetic tools have been developed [5]. Because of the genetic accessibility and the ease of its handling, a variety of new applications have been developed. Examples are the expression of cytokines and bacterial or viral antigens [6,7], enzymes [8], membrane proteins [9] and metabolic transformations [10]. These studies show that *L. lactis *is a suitable host for applications beyond its traditional use in food fermentations.

One of the crucial developments has been the construction of a food grade [11] and regulated gene expression system based on the regulation mechanism of the nisin A operon of *L. lactis*. In this operon the gene product nisin (a 34 amino acid bacteriocin) activates its own transcription at ng/ml amounts [12]. The elements of this regulatory system have been isolated and inserted in a suitable host strain, constituting the powerful regulated gene expression system NICE (NIsin-Controlled gene Expression system) [5,13]. The NICE system is widely used on laboratory scale for research and for over-expression of genes of interest [8,9,14]. However, experience in large scale application and fermentation of the NICE system is very limited.

Lysostaphin is a 25-kD antibacterial protein, produced by *Staphylococcus simulans *biovar. Staphylolyticus, that can hydrolyze the Gly-Gly bonds in the cell wall of the pathogens *S. aureus *and *S. epidermidis *and thus lyse these bacteria [15,16]. Lysostaphin has been proven to be an effective agent against the widespread hospital infectious agent *S. aureus *[17-19].

In the present study we describe for the first time a large-scale (3000 L) regulated gene expression process for the production of a heterologous protein – lysostaphin – in *L. lactis *using the NICE expression system. Furthermore, we also describe the purification of lysostaphin from the 3000-L fermentation batches resulting in a 90% pure pharmacological intermediate that has been further purified, formulated and used in clinical phase I studies.

## Results

### Construction of a lysostaphin expression system in *L. lactis*

**Figure 1:**
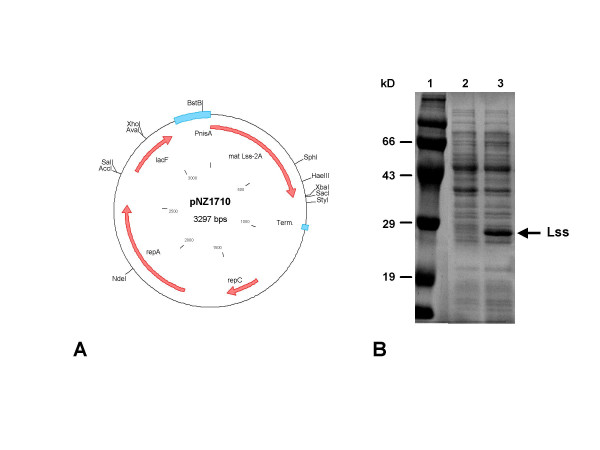
**Plasmid bearing the lysostaphin gene and over expression of this gene**. A, plasmid construct for nisin-controlled lysostaphin production. P_nisA_, nisin-controlled promoter; matLss-2A, coding sequence for mature lysostaphin lacking the first 2 alanine residues; Term., transcription terminator; *repC *and *repA*, replication genes; *lacF*, food-grade lactose selection marker. B, SDS-PAGE showing the intracellular production of lysostaphin upon induction with nisin. 1, molecular weight marker; 2, cell extract without nisin-induction; 3, cell extract with induction with 10 ng/mL nisin; Lss, lysostaphin

The coding sequence of the lysostaphin gene of *S. simulans *biovar. Staphylolyticus, lacking its first two alanine residues [15], was cloned after PCR amplification into the NICE vector pNZ8148, resulting in plasmid pNZ1709 (see Table 1). The obtained construct was verified by nucleotide sequencing. Subsequently, the chloramphenicol-resistance cassette was exchanged for the *lacF *gene of *L. lactis *[11], leading to pNZ1710 (Fig. 1A). In this plasmid expression of the lysostaphin gene *lss *is under control of the nisin-inducible *nisA *promoter. Furthermore, this plasmid is selected by a food-grade mechanism, i.e. growth on lactose, and does not contain an antibiotic-resistance gene or its remnants. Lysostaphin, a 25-kD antibacterial protein, was produced in the cytoplasm of the cells. Figure 1B shows an SDS-PAGE image of the intracellular soluble protein fractions of NZ3900 (pNZ1710) before and after induction with nisin. After induction with nisin, lysostaphin is accumulated in the cell to about 10% of the soluble protein fraction, as estimated from SDS-PAGE (Figure 1B). Lysostaphin was isolated, purified (see below) and its N-terminal amino acid sequence was determined to be Thr-His-Glu-His-Ser-Ala [15]. This indicates that the correct protein was produced, that no significant intracellular degradation occurred and that the N-terminal formyl-methionine residue was removed.

**Table 1:** Strains and Plasmids

**Bacterial strain/plasmid**	**Properties**	**Reference**
***Bacteria***		
*Lactococcus lactis *subsp. *cremoris *NZ3900	Integration of *nisRnisK *in the chromosome; integration of the *lac *operon in the in the chromosome and deletion of *lacF *resulting in a lactose- negative host strain that can be complemented by *lacF*	[25]
		
***Plasmids***		
pNZ8148	P_nisA_, Cm^R^; replicon of rolling circle plasmid pSH71, basic NICE vector, derivative of pNZ8048	[12] [26]
pNZ1709	Lysostaphin gene under control of P_nisA_, Cm^R^; derivative of pNZ8148	This work
pNZ1710	Lysostaphin gene under control of P_nisA_, *lacF*; expression vector for food- grade expression, selection on growth with lactose; derivative of pNZ1709	This work [11]

### Development of a fermentation medium and an induction scheme for lysostaphin production

In the development of human and animal pharmaceuticals it is important that the product is guaranteed BSE (Bovine Spongiform Encephalomyelitis) agent-free. All commercially available pre-formulated media for lactococci [e.g. M17 [20] contain components of animal origin such as meat extract and are possible sources for the BSE agent. Therefore, a new medium based on hydrolysed plant protein and yeast extract was developed. The key components of that medium are an entirely plant-based peptone and a yeast extract that is clear in a solution of at least 1%. The peptone chosen, was made from soy protein digested with the plant derived proteinase papain. Additionally, the medium contained 5% lactose (certified BSE free) to allow unlimited growth under pH-regulated growth conditions. Finally, Mg^2+ ^and Mn^2+ ^were added as known growth enhancers for lactic acid bacteria [20]. For details of the composition and sterilization of the medium see Methods.

**Figure 2:**
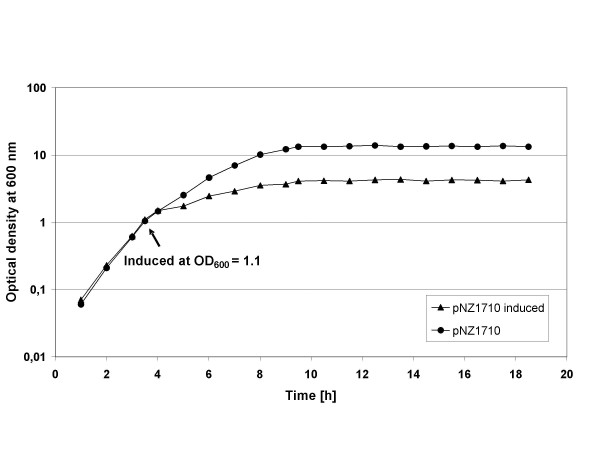
**Growth characteristics of 1-L culture**. Culture at 1-L scale of *L. lactis *NZ3900 containing the lysostaphin expression plasmid pNZ1710 with and without induction by 10 ng/mL nisin.

Induction of lysostaphin production was carried out at an optical density at 600 nm of about 1 (light path 1 cm) (mid exponential growth phase) (0.3 g/L cell dry weight [21]) by adding nisin (Figure 2). After induction, lysostaphin production proceeded for 6 – 8 hours. Figure 2 shows that upon induction, growth of the culture is severely inhibited. This is likely the result of lysostaphin accumulation in the cell that appears to have growth inhibiting properties (viable plate counts drop within 20 min after induction 3–4 orders in magnitude).

### Scale-up of lysostaphin production to 300 L and 3000 L

**Figure 3:**
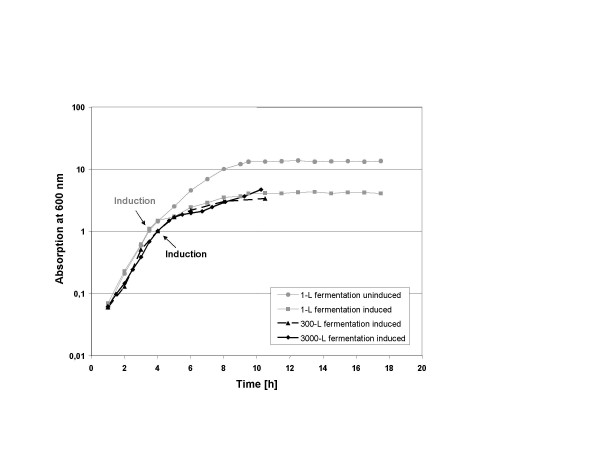
**Comparison of growth characteristics of 1-L, 300-L and 3000-L cultures**. Culture of *L. lactis *NZ3900 containing the lysostaphin expression plasmid pNZ1710 at 1-L, 320-L and 3000-L scale with induction by 10 ng/mL nisin. As comparison, growth of an uninduced culture at 1-L scale is shown.

Lysostaphin production was scaled up from 1-L scale to 300-L scale and eventually 3000-L fermentations. Growth and induction conditions found at laboratory scale were directly transferred to the 300-L and 3000-L scale: Induction at an optical density OD_600 _= 1 with 10 ng/ml nisin. Details of media preparation for the larger scales are described in Methods. Figure 3 shows the growth characteristics of induced cultures of *L. lactis *carrying the plasmid pNZ1710 at all three scales. All three cultures behaved very similarly, despite the difference in scale of more than 3 orders of magnitude. The lysostaphin yields of the 1-L and 3000-L fermentations after induction were approximately 100 mg/L. Four consecutive 3000-L production runs were carried out to produce raw material for further down stream processing. The growth characteristics of the four fermentation runs were virtually identical. In each run approximately 300 g lysostaphin (100 mg/L) were produced and subsequently used as starting material for purification.

### Downstream processing of the 3000-L lysostaphin production batch

A downstream processing protocol was designed for the preparation of a pharmaceutical intermediate that could be used for further purification and formulation to carry it into clinical phase I trials.

The basic operations were concentration and washing of the cells, destruction of the cells by homogenization, removal of the cell debris, and capturing of the lysostaphin by chromatography.

The fermenter content was concentrated about 20-fold by filtration (tangential flow) over a 0.8 μm ceramic membrane (3.8 m^2^) and then 200% diafiltrated to remove most of the residual medium components. The retentate was subjected to continuous homogenization at 1400 bar, 80 L/h. The homogenization procedure was repeated three times to ensure complete release of the intracellular lysostaphin produced.

The cell debris was then separated from the intracellular fraction by filtration (tangential flow) over a 0.8 μm ceramic membrane (3.8 m^2^). The homogenate was first concentrated to 100 L and then 300 % diafiltrated to wash out any lysostaphin associated with the cell debris. The resulting lysostaphin-containing filtrate of about 400 L was stored frozen before loading onto the chromatography column.

### Capturing lysostaphin

**Figure 4:**
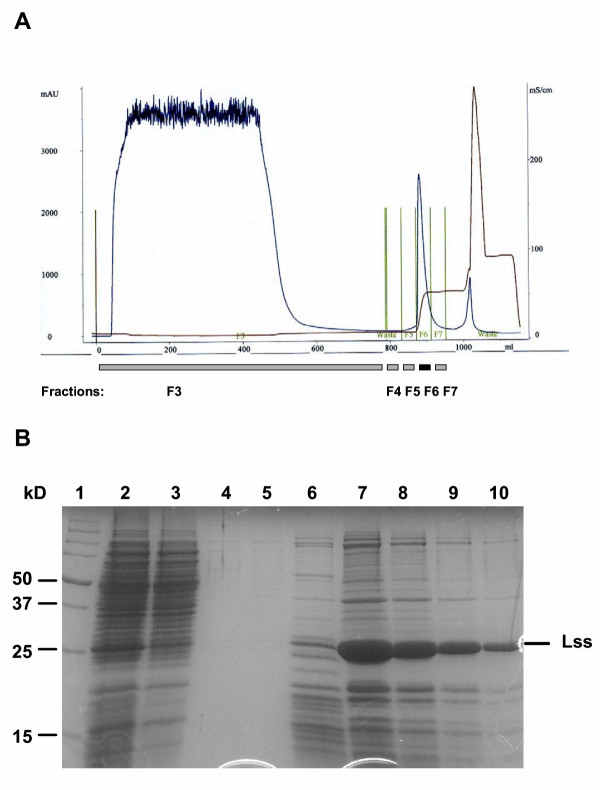
**Purification of the overproduced lysostaphin**. A, Typical chromatogram of a lysostaphin capture step. NaCl concentration (brown line) and absorption at 280 nm (blue line) are indicated. Fractions that were analysed by SDS-PAGE are indicated by grey bars underneath. The lysostaphin fraction (F6) is indicated with a black bar. B, SDS-PAGE analysis of the different fractions of the capture chromatography. 1, molecular weight marker; 2, cell extract before loading; 3, flow-through fraction (F3); 4, 5 and 6, fractions F4, F5 and F7; 7, lysostaphin fraction F6; 8, 9 and 10, fraction F6 diluted 1:2, 1:4 and 1:8.

A cation-exchange chromatography capture step was selected based on the relatively alkaline isoelectric point (pH 9.5) of lysostaphin [16]. Because of superior performance, the strong exchanger SP-Sepharose FF was chosen over the weak exchanger CM-Sepharose FF. Optimum lysostaphin binding was found at pH 7.5 in phosphate buffer. Lysostaphin was eluted using a NaCl step gradient at 0.5 M NaCl with the same pH and phosphate buffer concentrations (Methods) as used for loading (Figure 4A and 4B show an elution profile and SDS-PAGE analysis). Since maximum binding of lysostaphin was hindered by unknown components in the cell extract, the flow-through was re-fed to the column twice to capture more than 90% of lysostaphin from the cell extract. Finally, the eluate was diluted and all captured lysostaphin was applied to the column at once for an additional recapture step (Methods). The resulting material was ca. 90% pure lysostaphin as determined by SDS-PAGE analysis (Methods). The lysostaphin production yield in the fermentation was about 100 mg/l. Therefore about 300 g lysostaphin had been produced in each 3000-L fermentation run. The mean total yield of the downstream process was about 120 g, resulting in 40% recovery of the originally produced lysostaphin.

## Discussion

Nisin-regulated gene expression in *Lactococcus lactis *has been shown to be an effective and multifunctional tool [2,5,7,9]. *L. lactis *has numerous characteristics that make it an interesting host for industrial-scale heterologous protein production: it is 100% food grade, including plasmid selection systems, it produces no endotoxins or other toxic substances, it produces no inclusion bodies and no spores, it is grown in simple non-aerated, stirred fermentations and produces no extracellular proteinases. With these characteristics, it can be used for food applications, as a source of enzymes, for metabolic transformations and for the production of biologicals. The fact that *L. lactis *is food grade also means that cellular components or enzymes can be used with only partial purification or directly in the crude cell extract without further purification. Despite 10 years of laboratory use of the NICE system, we are not aware of any reports that describe the step to large-scale industrial application of this tool. This paper describes the successful development and scale-up of a process for the production of a heterologous protein using lysostaphin as an example. Lysostaphin is an antibacterial protein from *S. simulans *biovar. Staphylolyticus, that has potential in topical and systemic applications for the treatment of *Staphylococcus aureus *infections [17-19]. Lysostaphin is a clear example for the benefits of a regulated expression system, since constitutive intracellular lysostaphin expression leads to rapid cell death. Another advantage of regulated gene expression is that the cells can be pre-grown to a certain cell density before the energetically costly production of a foreign protein is switched on.

Furthermore, a food-grade plasmid selection system based on lactose consumption has been used, making the use, detection and tracing of antibiotics unnecessary. This food-grade construct allowed the production of a pharmaceutical intermediate in a cheaper food-grade production plant rather than in a cGMP facility. Similarly, such a process could be used to make other industrially interesting intermediates such as enzymes for food and (bio)chemical applications, probiotic preparations, etc.

The process described in this paper was first developed at 1-L scale and subsequently transferred to the 300-L and 3000-L scale. This scale-up was without problems, without specific calculations and without changes in the available equipment. *L. lactis *is a simple fermentative, oxygen-tolerating bacterium converting lactose or glucose into lactic acid. Therefore, no oxygen transfer is needed during the fermentation. The only condition that needs to be met is appropriate mixing of the whole culture to ensure evenly distributed nutrients and effective distribution of nisin for the induction of gene expression. One significant difference exists in the addition of nisin to the culture at different scales. At laboratory scale this addition takes a few seconds, while at 3000-L scale it takes 2 – 5 min. Despite the difference in addition times, no adverse effects on the induction process and lysostaphin production were observed.

We carried out four consecutive fermentations at the 3000-L scale. The growth of the culture in all four fermentation runs was nearly identical, as was the final yield of lysostaphin after purification (about 120 g per batch), indicating the biological and technical robustness of the process.

The down-stream processing shows that *L. lactis *can effectively be separated from the fermentation medium and that the cell content can be released by high-pressure continuous homogenization. In the present process three passages for complete destruction of the cells are used. Preliminary results show that two passages may be enough. The cell debris can simply be removed by a second filtration step in the same equipment that is used for the cell separation. After separation of the cell content from the debris, the product can be used as crude extract with enriched enzyme activity or different routes can be followed to further purify or enrich the active component: ultrafiltration, selective precipitation and chromatography. In the present process, lysostaphin was captured on SP-Sepharose FF in two consecutive steps. We found that an as yet unknown component in the cell extract hampered binding of lysostaphin to the resin. This obstacle was overcome by repeated loading of the flow-through to the same column. The solution of captured lysostaphin apparently lost the interfering component and could be completely bound to the resin in one recapture run. Further research is needed to identify the interfering compound and to design a process in which lysostaphin can be captured in a single run.

While 100 mg/L yield is relatively low for bulk productions, it may be acceptable for specialized productions. Careful optimization of growth and induction can lead to considerable yield increases. This was also demonstrated for lysostaphin were in a separate set of experiments lysostaphin production was optimized and a yield of 300 mg/L was reached [22]. One of the challenges is the development of a fermentation process in which this fermentative organism can be grown to higher cell densities.

The downstream process as a whole consists only of a few steps and is thereby simple, fast and highly reproducible. After further optimization it could easily be carried out within 48 h after the fermentation run, limiting the whole process to one week from seed culture to product.

## Conclusion

The present publication describes for the first time that it is possible to use the lactic acid bacterium *L. lactis *for industrial scale heterologous protein production. Furthermore, we demonstrate that the widely used nisin-controlled gene expression system NICE is fully operative at this scale. This opens the way for a food grade alternative expression system to the commonly used host *E. coli*. Other advantages are that *L. lactis *does not produce endotoxins or inclusion bodies, and does not produce spores and extracellular proteinases. This opens up a wide range of pharmaceutical, cosmetics, biochemical, food, and feed applications.

## Methods

### Bacterial strains and plasmids

Table 1 shows the strain and the plasmids used in this study. The bacteria were maintained as frozen stock at -80°C.

### Growth media and cultivation conditions

For the genetic construction work the bacteria were routinely grown in M17 medium [20] fortified with 1% glucose or 0.5% lactose and 5 μg/ml chloramphenicol for selection on chloramphenicol resistance.

For 1-L, 300-L and 3000-L fermentations the following medium was used: 5% lactose (pharmaceutical-grade lactose Lactochem 207, Borculo DOMO Ingredients, Zwolle, The Netherlands), 1.5% peptone from soy (VWR-Merck, product number 111932, Amsterdam, The Netherlands), 1% yeast extract (BioSpringer, product number 1105C0/180, Maisons Alfort, France), 1 mM MgSO_4 _(VWR-Merck), 0.1 mM MnSO_4 _(VWR-Merck). For the 1-L scale all ingredients were dissolved in water and the medium was sterilized for 20 min at 110°C. For the 300- and 3000-L scale all ingredients were dissolved in water and subsequently stream-sterilized (Crepaco, Bryan, Texas, U.S.A.) for 20 s at 140°C (batch-wise sterilization will cause chemical reactions in the medium that will inhibit growth of the bacteria). Bacteria were inoculated at 1 % and grown at 30°C. For 300- and 3000-L fermentations inoculum was prepared as follows. 2 mL frozen stocks were removed from -80°C freezer, inoculated into 100 and 300 ml fermentation medium, respectively, and grown under acidifying conditions as standing culture for 16 h. For the 300-L fermentation, 30 ml of this culture were inoculated into 3 L fermentation medium and cultivated under acidifying conditions as standing culture for 16 h. For the 3000-L fermentation, 300 mL were inoculated into 30 L fermentation medium in a 75-L fermentor and cultivated for 16 h with a stirring speed of 50 rpm under acidifying conditions.

### Nisin induction

0.04% nisin powder (Sigma-Aldrich Chemie, Zwijndrecht, The Netherlands) was dissolved in 0.05% acetic acid and precipitated proteins were removed by centrifugation. Cells were grown to an optical density at 600 nm of 1 (light path 1 cm) (= 0.3 g/L cell dry weight [21]) at which point 10 ng/ml of nisin (final concentration) was added. Following the induction, the culture was incubated for 6 – 8 h before harvest. The 3000-L fermentations were terminated by rapid cooling to 4°C and stored for about 12 h at this temperature before further processing.

### Molecular techniques

Standard genetic techniques were carried out according to Sambrook et al. [23]. SDS-PAGE was performed according to Laemmli [24]. N-terminal amino acid sequencing was outsourced (Protein Sequencing Laboratories, University of Leiden, Netherlands).

### Detection and quantification of lysostaphin

Lysostaphin production was routinely monitored using SDS-PAGE. The activity of the enzyme was determined using the *Staphylococcus carnosus *cell wall degradation assay [15]. Lysostaphin was quantified using capillary electrophoresis as described in Mierau et al. [22]. Purity of lysostaphin was determined from scanning and analysis of SDS-PAGE gels with a PowerLookIII scanner (UMAX Systems GmbH, Düsseldorf, Gemany) and the ImageMaster software (GE Healthcare, formerly Amersham Biosciences, Rosendaal, The Netherlands).

### Equipment for fermentation, filtration and homogenization

For 1-L, 30-L, 300-L, and 3000-L fermentations, stirred, temperature- and pH-regulated tank fermentors were used (1 L: Applicon fermentor with Biocontroller ADI 1030, Applicon, Frederiksberg, Denmark; 30L and 300 L: Chemap fermentors, Chemap AG, Volketswil, Switzerland; 3000 L: custom made fermentor). Stirring was carried out with a propeller blade stirrer at approximately 50 rpm, to ensure proper mixing of the base and of nisin. During the fermentation, temperature and base consumption were recorded to monitor the process. To determine the cell density of the cultures and to find the time point for induction, samples were taken at regular intervals and the OD at 600 nm (light path 1 cm) was determined.

For microfiltration, a one-stage filtration installation was used with a Ceraver ceramic membrane of 0.8 μm pore size and 3.8 m^2 ^surface (Membralox, Pall, East Hills, New York, U.S.A.). The flow rate was approximately 330 L/h.

Concentrated cell suspensions were disintegrated using a continuous homogenization process at 1400 bar with a flow rate of about 80 L/h (Homogenizer 10.51 VH, APV, Hendrik Ido Ambacht, The Netherlands). To prevent overheating, the pressure-reduction nozzle was cooled with ice water.

### Chromatography

Large-scale chromatography was carried out with a Bioprocessor (max. flow rate of 120 L/h) and a BPG300 column (Amersham Biosciences, Roosendaal, The Netherlands). For the separation 14.1 L Sepharose Fast Flow resin (17-0792-04, Amersham Biosciences) was used, resulting in a bed height of 20 cm. The sample was prepared for chromatography by adjusting the pH to 7.2 with 0.4 M NaH_2_PO_4 _pH 7.5. The following buffers and cleaning solutions were used: Equilibration buffer, 50 mM NaH_2_PO_4 _pH 7.5; Elution buffer, 50 mM NaH_2_PO_4 _+ 0.5 M NaCl pH 7.5; Regeneration buffer, 50 mM NaH_2_PO_4 _+ 1 M NaCl pH 7.5; Cleaning solution, 1 M NaOH. A standard chromatography run was carried out as follows. The column was equilibrated with 2 volumes equilibration buffer at 120 L/h, the pH-adjusted sample was loaded at 100 L/h, the loaded column was washed with 3 volumes equilibration buffer at 120 L/h, lysostaphin was eluted with 2 column volumes elution buffer at 120 L/h and the column was cleaned with 1 column volume cleaning solution at 50 L/h. Finally, the column was regenerated with 2 column volumes regeneration buffer at 120 L/h. This process was repeated for all subsequent runs. Since the whole sample of about 400 L could not be loaded at one time (Results), it was divided into four portions of approximately 100 L each.

For recapture, the following buffers and solutions were used: Equilibration buffer, 12.5 mM NaH_2_PO_4 _+ 75 mM NaCl pH 7.0; Elution buffer, 25 mM NaH_2_PO_4 _+ 0.25 M NaCl pH 7.0, Cleaning solution and regeneration buffer were as mentioned above. For recapture, the eluate of the capture step was desalted using diafiltration and the pH was adjusted to pH 7.0. The column was equilibrated with 2 column volumes of equilibration buffer at 120 L/h, the sample was loaded at 100 L/h, the column was washed with 3 volumes equilibration buffer at 120 L/h and the product was eluted with 2 column volumes elution buffer. Cleaning and regeneration was done as described above.

## Authors' contributions

IM and JM set up and supervised the project. IM was in charge of the genetic work. ES and BB worked out and were in charge of the fermentations. PL set up and was in charge of the filtration processes. IvS and EF set up and carried out the chromatography steps.
